# Osteosynthesis of an acromion fracture using a proximal clavicle plate: a case report

**DOI:** 10.1093/jscr/rjag805

**Published:** 2026-09-11

**Authors:** Yuki Yamai, Ryogo Furuhata, Atsushi Tanji, Noboru Matsumura

**Affiliations:** Department of Orthopaedic Surgery, Ashikaga Red Cross Hospital, 284-1 Yobe-cho, Ashikaga-shi, Tochigi, 326-0843, Japan; Department of Orthopaedic Surgery, Ashikaga Red Cross Hospital, 284-1 Yobe-cho, Ashikaga-shi, Tochigi, 326-0843, Japan; Department of Orthopaedic Surgery, Ashikaga Red Cross Hospital, 284-1 Yobe-cho, Ashikaga-shi, Tochigi, 326-0843, Japan; Department of Orthopaedic Surgery, Keio University School of Medicine, Shinjuku-ku, Tokyo, 160-8582, Japan

**Keywords:** acromion fracture, plate fixation, clavicle plate, outcome

## Abstract

Displaced acromion fractures are commonly treated surgically; however, no implants have been specifically designed for this fracture type. Although various plates have been used, fixation failure and implant-related irritation remain concerns. We report a case of acromion fracture fixation using a proximal clavicle plate. A 77-year-old man presented with right shoulder pain after a fall. Computed tomography revealed an Ogawa type III acromion fracture extending to the spinoglenoid notch. Through a posterior approach along the scapular spine, the fracture was anatomically reduced and fixed with a proximal clavicle locking plate. Four screws, including two longer than 30 mm, were inserted medially to the fracture site, and six screws were inserted laterally. Neither fixation failure nor implant-related pain occurred, and complete bone union was achieved. Fixation with a proximal clavicle plate may represent a useful treatment option for acromion fractures, particularly in cases with highly variable acromial and scapular spine anatomy.

## Introduction

Traumatic acromion fractures are rare, accounting for <1% of all fractures [1]. Displaced acromion fractures are associated with a high risk of symptomatic nonunion [2]; therefore, surgery is often indicated. Surgical procedures for acromion fractures include tension band wiring, hollow screw fixation, and plate fixation; however, owing to its rarity, there is no established gold-standard surgical procedure. Although plate fixation is considered to provide more rigid fixation than tension band wiring and screw fixation [3], no plates are specifically designed for acromion fractures and various types of plates have been used [2–11]. However, implant failure [2, 7, 8] and plate irritation [2, 3, 10, 11] can occur postoperatively.

We report a case of acromion fracture fixation using a proximal clavicle plate. To our knowledge, this is the first report describing the use of a proximal clavicle plate for fixation of an acromion fracture.

## Case report

**Figure 1:**
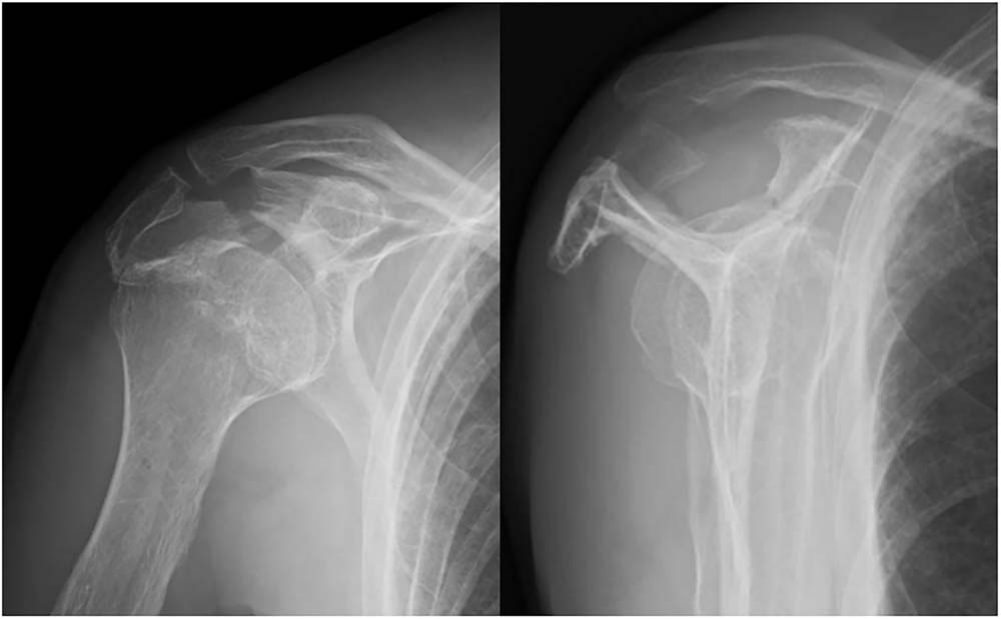
Preoperative radiographs of the right scapula showing displaced acromion and coracoid process fractures.

**Figure 2:**
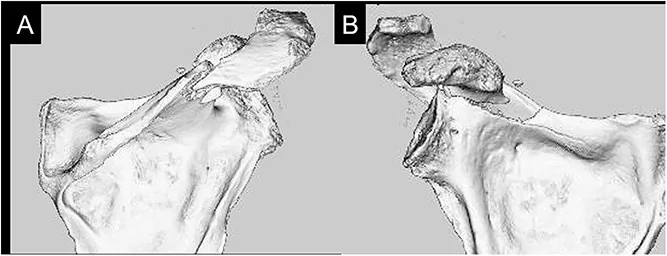
CT of the right scapula. 3D-CT demonstrates an Ogawa type III acromion fracture with a fracture line extending to the spinoglenoid notch (A) and an Ogawa type I coracoid process fracture (B).

A 77-year-old man presented to our hospital with persistent right shoulder pain since a fall while climbing a mountain 3 days earlier. He had no significant medical history. He had difficulty moving his shoulder because of severe pain. Radiography showed displaced fractures of the acromion and coracoid process (Fig. 1). Computed tomography (CT) revealed an Ogawa type III acromion fracture [12] with a fracture line extending to the spinoglenoid notch (Fig. 2A) and an Ogawa type I coracoid process fracture [13] (Fig. 2B). Surgery was indicated because of disruption of the superior shoulder suspensory complex.

**Figure 3:**
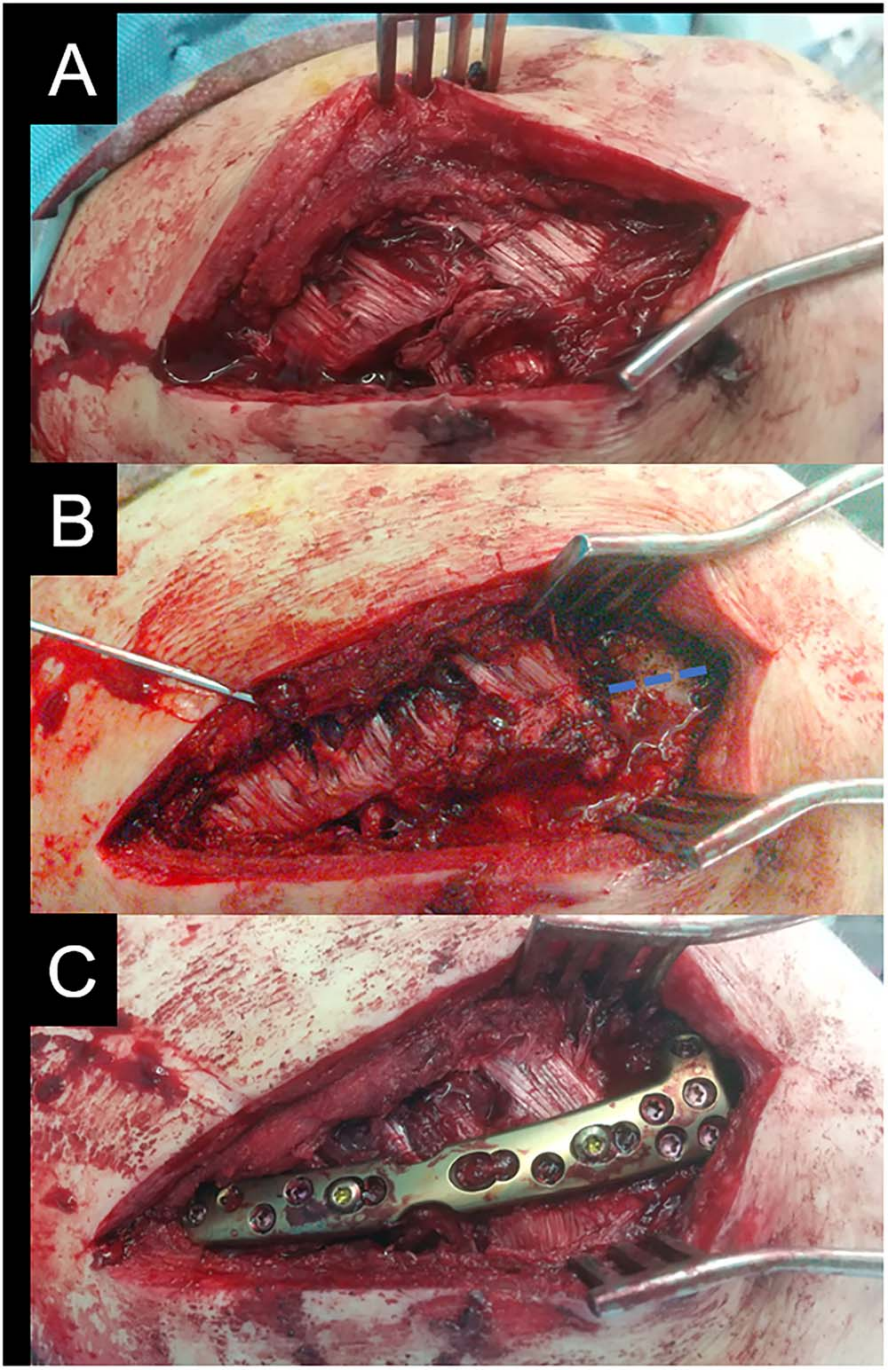
Intraoperative findings during osteosynthesis of the right acromion fracture. Using a skin incision along the scapular spine, a displaced acromion fracture is identified (A). Fracture fragments are reduced, and a Kirschner wire is placed to temporarily fix the fragments (B). After detaching 2 cm of the trapezius muscle attached to the acromion angle subperiosteally (dashed line), a proximal clavicle plate is placed on the dorsal border of the scapular spine (C).

**Figure 4:**
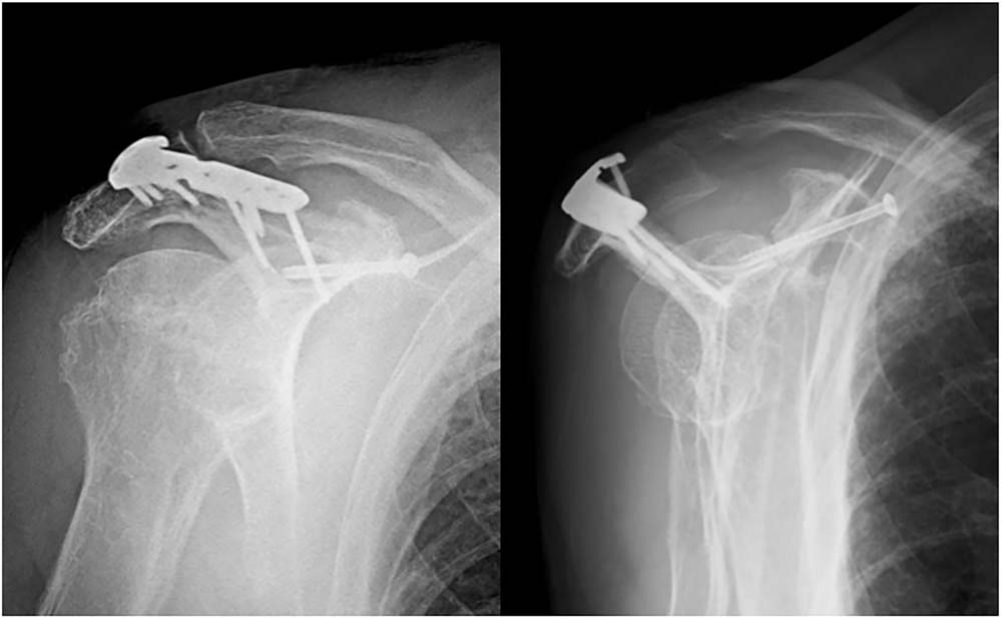
Postoperative radiographs of the right scapula obtained immediately after surgery.

Surgery was performed with the patient in the left lateral recumbent position. For the acromion fracture, we made a skin incision along the scapular spine, exposing the fracture site (Fig. 3A). We reduced the fractured fragments and temporarily stabilized them with a Kirschner wire (Fig. 3B). After detaching 2 cm of the trapezius muscle attached to the acromion angle subperiosteally, we placed a proximal clavicle plate (VA-LCP® Clavicle Plate, Synthes, Oberdorf, Switzerland) on the dorsal border of the scapular spine. Because of the good anatomical fit of the plate to the scapular spine, only minor plate bending was required. We inserted one cortical screw and three locking screws medial to the fracture site, with the screw trajectories directed perpendicular to the scapular spine. Two of the four screws had lengths >30 mm. One cortical screw and five locking screws were inserted lateral to the fracture site (Fig. 3C). For the coracoid process fracture, we used a 4.0 mm-cannulated cancellous screw (MDM, Tokyo, Japan) for reduction and fixation (Fig. 4).

The affected arm was placed in a sling for 3 weeks. From 1 week postoperatively, active shoulder range-of-motion exercises and rotator cuff strength exercises within ˂90° of shoulder elevation were commenced. Full shoulder range-of-motion exercises were initiated 5 weeks postoperatively. Scapular stabilization exercises were initiated 9 weeks postoperatively.

**Figure 5:**
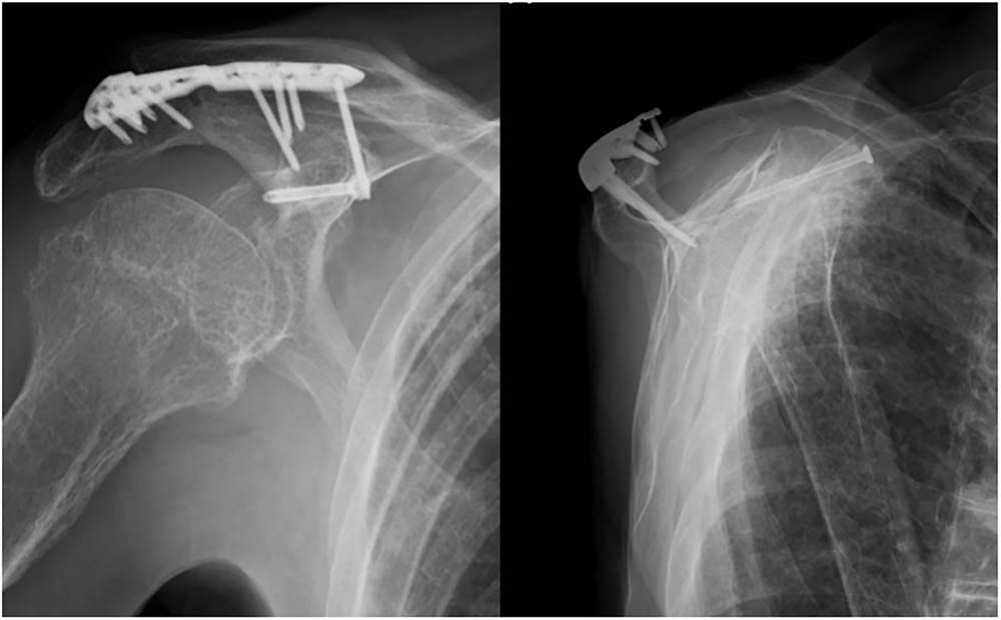
Postoperative radiographs of the right scapula obtained 1 year after surgery.

Radiography performed 1 year postoperatively confirmed bone union without displacement of the fracture fragment (Fig. 5). The patient had no plate-related pain, and the range of shoulder motion (right/left) was 130/140° for anterior elevation and 30/40° for external rotation. The Constant score was 89 at 1 year postoperatively.

## Discussion

In the present case, osteosynthesis using a proximal clavicle plate achieved solid bone union and satisfactory shoulder function without postoperative complications.

Plate fixation using straight plates [2–4, 6–8, 11], distal clavicle plates [3, 9–11], distal humerus plates [5, 8], and olecranon plates [8] has been reported for acromion fractures; however, the application of plates for proximal clavicle fractures has not been reported. Fixation using other plates has provided relatively good surgical outcomes [2, 4–9]. However, because of substantial anatomical variation in the scapular spine [14], implant failure [2, 7, 8] and plate irritation [2, 4, 14, 15] resulting from implant-anatomy mismatch have been reported [10]. In this case, these complications were not observed, and the postoperative functional score was comparable to that reported in previous studies [5, 6], suggesting that a proximal clavicle plate represent a useful alternative when conventional implants do not adequately match the highly variable anatomy of the acromion and scapular spine.

The proximal clavicle plate offered two additional advantages beyond its favorable anatomical fit. First, the placement of the proximal clavicle plate on the scapular spine enabled the insertion of sufficiently long screws that reached the anterior cortex of the scapular body. A previous biomechanical study showed that placing the locking plate on the dorsal subcutaneous border of the scapular spine reliably allowed longer screws, resulting in a strong fixed-angle construct medially, compared with plate placement on the supraspinatus fossa [15]. Another case series suggested that a medially extended plate and optimized screw fixation are important for preventing implant-related complications [11]. In this case, four screws (two >30 mm) were inserted medially to the fracture site, which may have contributed to the rigid fixation. Second, the proximal clavicle plate enabled the lateral insertion of a sufficient number of locking screws while minimizing the disruption of the deltoid and trapezius origin from the acromion angle. The lateral acromion is characterized by reduced thickness and a thin cortical shell; therefore, lateral fixation of the acromion is challenging [8]. The insertion of a sufficient number of locking screws in the lateral acromion presumably improves fixation strength [9, 10]. However, achieving such lateral fixation often requires extensive dissection of the deltoid and trapezius insertions at the acromial angle, particularly when distal clavicle plates are used. In this case, we were able to insert six screws lateral to the fracture site, maintaining a trapezius detachment at 2 cm, suggesting that the muscle dissection from the acromion angle was relatively minimal.

This case report highlights the potential utility of a proximal clavicle plate for fixation of acromion fractures. Osteosynthesis with a proximal clavicular plate can achieve anatomical reduction and rigid fixation of acromion fractures, with satisfactory postoperative outcomes. Proximal clavicle plate fixation may be a useful option for acromion fractures, particularly in patients with highly variable acromial and scapular spine anatomy.

However, the proximal clavicle plate is not indicated for Ogawa type I acromion fractures because plate placement along the anterior aspect of the acromion is difficult. Moreover, this report is limited by its single-case design and the absence of biomechanical comparison with other fixation methods. Further studies are needed to evaluate the reproducibility and broader applicability of this technique.

## Data Availability

Data supporting the findings of this study are available from the corresponding author upon request.
